# Hypoxia and hypoxia mimetics differentially modulate histone post-translational modifications

**DOI:** 10.1080/15592294.2020.1786305

**Published:** 2020-07-01

**Authors:** Kuo-Feng Hsu, Sarah E. Wilkins, Richard J. Hopkinson, Rok Sekirnik, Emily Flashman, Akane Kawamura, James S.O. McCullagh, Louise J. Walport, Christopher J. Schofield

**Affiliations:** a Chemistry Research Laboratory, Department of Chemistry, University of Oxford, Oxford, UK; b Tri-Service General Hospital, National Defense Medical Center, Taipei, Taiwan; c Leicester Institute of Structural and Chemical Biology and School of Chemistry, University of Leicester, Leicester, UK; d Radcliffe Department of Medicine, Division of Cardiovascular Medicine, BHF Centre of Research Excellence, Wellcome Trust Centre for Human Genetics, Oxford, UK; e Chemistry - School of Natural and Environmental Sciences, Newcastle University, UK; f Protein-Protein Interaction Laboratory, The Francis Crick Institute, London, UK; g Department of Chemistry, Molecular Sciences Research Hub, Imperial College London, London, UK

**Keywords:** Hypoxia, histone post-translational modifications, epigenetics, hypoxia mimetics, epigenetics, HIF, intact protein mass spectrometry, iron chelating drugs, 2-oxoglutarate/α-ketoglutarate oxygenases

## Abstract

Post-translational modifications (PTMs) to the tails of the core histone proteins are critically involved in epigenetic regulation. Hypoxia affects histone modifications by altering the activities of histone-modifying enzymes and the levels of hypoxia-inducible factor (HIF) isoforms. Synthetic hypoxia mimetics promote a similar response, but how accurately the hypoxia mimetics replicate the effects of limited oxygen availability on the levels of histone PTMs is uncertain. Here we report studies on the profiling of the global changes to PTMs on intact histones in response to hypoxia/hypoxia-related stresses using liquid chromatography-mass spectrometry (LC-MS). We demonstrate that intact protein LC-MS profiling is a relatively simple and robust method for investigating potential effects of drugs on histone modifications. The results provide insights into the profiles of PTMs associated with hypoxia and inform on the extent to which hypoxia and hypoxia mimetics cause similar changes to histones. These findings imply chemically-induced hypoxia does not completely replicate the substantial effects of physiological hypoxia on histone PTMs, highlighting that caution should be used in interpreting data from their use.

## Introduction

In response to hypoxia in animals, adaptive processes take place in cells and tissues that work to maintain oxygen supply and/or increase the efficiency of its use. Such processes include erythropoiesis, angiogenesis, and the partial shutdown of high-energy consuming processes[1]. Many of these responses involve changes in expression patterns, as mediated by the hypoxia-inducible transcription factor isoforms [2,3]. In humans, hypoxia-inducible factor-α (HIF-α) is regulated by two types of 2-oxoglutarate- (2OG)/Fe(II)-dependent oxygenases: the HIF prolyl hydroxylases (PHD1, PHD2 and PHD3) and the asparaginyl hydroxylase, factor inhibiting HIF (FIH) [1,4–6]. In hypoxia, reduced oxygen availability limits catalysis by the HIF hydroxylases, which promotes HIF target gene transcription, both by stabilizing HIF-α and by hindering its interaction with the histone acetyltransferases CBP/p300.

Another layer of hypoxic gene regulation is provided by histone post-translational modifications (PTMs) [7–9]. Different patterns of histone PTMs are proposed to regulate the expression of different sets of HIF target genes in a context-dependent manner[10]. Many of the enzymes responsible for modifying these PTMs are themselves HIF target genes (e.g. G9a, KDM3A), and thus their levels, and thereby activity, are also regulated by oxygen availability [9,11]. Furthermore, some histone-modifying enzymes are directly dependent on oxygen for catalytic activity, adding an additional potential for hypoxic regulation [12–16]. The transcriptional response to hypoxia and its regulation is therefore both comprehensive and highly complex.

Histone demethylation is mediated by two families of enzymes. Like the HIF hydroxylases, the larger of these families, the JmjC histone lysine demethylases (KDMs), are 2OG/Fe(II)-dependent oxygenases, which require oxygen as an essential cosubstrate to catalyse histone demethylation [17,18]. As such, their enzymatic activity has the potential to be compromised by hypoxia [12–16]. On the other hand, transcriptional upregulation of specific JmjC KDMs is reported to be mediated by HIF[11]. Increasing evidence suggests that hypoxia has the potential to induce global changes in histone methylation both through directly compromising the enzymatic activity of JmjC KDMs and by altering the expression levels of JmjC KDMs and lysine methyltransferases.

Iron also plays a critical role in the activities of the PHDs and FIH and in some contexts its availability is proposed to regulate HIF signalling[19]. Iron chelators are commonly used to mimic hypoxia by inhibiting PHDs and FIH (and likely other 2OG oxygenases), so hindering HIF-α degradation/inhibition of HIF transcription [20,21]. Iron chelators such as desferrioxamine (DFO, Desferal^®^), deferiprone (CP20) and deferasirox (Exjade) are used clinically to treat human iron overload diseases (Supplementary Figure S1) [22,23].

Unlike iron chelators, dimethyloxalylglycine (DMOG) is a cell-permeable 2OG analogue which acts as a broad-spectrum inhibitor of many 2OG/Fe(II)-dependent oxygenases[24]. DMOG treatment stabilizes HIF-α in normoxia, likely predominantly via inhibition of the HIF-α prolyl hydroxylases (PHDs), and it is consequently used as a hypoxia-mimetic agent [24,25]. Cobalt chloride (CoCl_2_) is also used to create hypoxia-mimetic stress by inducing the accumulation of HIF-α, likely in part via inhibition of the PHDs[26]. More specific PHD inhibitors, such as IOX2 and FG4592 (Roxadustat, which has been approved for use in some countries and is in late stage clinical trials in others for treatment of renal anaemia) [27,28], are used to replicate aspects of reduced oxygen availability on PHD activity. Hypoxia and hypoxia mimetics are reported to affect epigenetic regulation by changing the activities of histone modifying enzymes and the levels of HIF isoforms [8,12,29,30]. However, differences between the effects of hypoxia and hypoxia mimetics on the levels of histone PTMs have not been determined.

Mass spectrometry (MS) is increasingly used to investigate chromatin/chromatin-modifying enzymes [31–33]. MS-based identification of PTMs on intact histones (a ‘top-down’ approach) can provide insights into combinatorial modification patterns[34]. Coupled with proteolytic digestion (a ‘bottom-up’ approach), MS is a useful method for elucidating the exact positions of PTMs observed on histones[35]. However, although protocols have been developed, the quantitative interpretation of proteomic MS data on heavily modified histone tails is challenging [31,36,37].

We have thus been interested in pursuing liquid chromatography-MS (LC-MS) as a relatively simple method for separating the different core histones (histones H3, H4, H2A and H2B) and simultaneously profiling their intact PTM status. Whilst previous studies have demonstrated the possibility of using LC-MS to study the global PTM status of core histones [38–40], there have been relatively few reports investigating these changes in the context of cellular stimuli [41–43].

Here we report a rapid, reliable LC-MS method to profile intact histone proteins and use it to compare the effects of hypoxia, and commonly used hypoxia mimetics, including iron chelators, PHD inhibitors and CoCl_2_, on the PTM profiles of intact histones. The results provide insights into the profile of histone PTMs in hypoxia and imply that the influences of hypoxia and so-called ‘hypoxia mimetics’ on epigenetic changes are not identical. These insights have implications of relevance for the development of new approaches to transcriptional regulation and clinical studies.

## Results

### MS profiling for histones analysis under optimized LC conditions

Based on previous procedures [38,43,44], chromatographic conditions were optimized to enable rapid and efficient separation of intact histones from human cells. Based on a desired combination of sufficient histone separation and a reasonably rapid run time, a 20 minute gradient was selected for our subsequent studies (Supplementary Figures S2,3). Although co-elution of histones H2B and H4 was observed, LC-MS enabled identification of H2B and H4 due to their mass differences (Supplementary Figure S4). As previously observed[40], most histone PTMs, e.g. methylation (+14 Da), acetylation (+42 Da) and phosphorylation (+80 Da), appear to have only small effects on the chromatographic behavior of individual proteins, and thus modified histone isoforms tend to co-elute with their unmodified precursors. Provisional assignments for the observed peaks and mass shifts were made based on histone variants identified to date (Supplementary Tables S1-6).

The interpretation of results in the context of intact histone protein molecular weight measurements is complicated by near mass redundancies, such as for trimethylation (+42.0470 Da) and acetylation (+42.0106 Da). To simplify discussion of the results, in description of mass shifts we therefore use the term methyl equivalent (m.e.) corresponding to a mass shift of 14 Da, though note 3 m.e. also corresponds to 1 acetylation, and hence multiple m.e. mass shifts do not necessarily correlate to methylation events. Mass shifts for H3.1 are given relative to the value for the unmodified protein (minus *N*-terminal methionine), and for H4 relative to the *N*-terminally acetylated protein. Note also that the intact histone method does not inform on positions of PTMs and that, in most cases, each modified peak (e.g. +1 m.e.) will correspond to a collection of histones, each singly modified, but at different residues. Tentative assignments (e.g. *N*-terminal acetylation and certain phosphorylation sites) are made on the basis of previous reports[45].

Cells were treated with compounds (Supplementary Figure S1) for varied time periods, before histone extraction followed by LC-MS analysis. To investigate the range of concentrations that could be used without decreases in cell viability, following compound treatment, membrane integrity was assessed using a trypan blue exclusion assay (Supplementary Figure S5)[46]. Our extraction and LC-MS protocol manifested low variability based on analyses of all core histone variants and their PTMs (Supplementary Figure S6), and was feasible with different cell types: histones from HeLa (human cervical carcinoma), RKO (human poorly differentiated colon carcinoma) and MCF-7 (human breast adenocarcinoma) cells were analysed (Supplementary Figure S7).

To validate the protocol, we conducted studies with an HDAC inhibitor, Vorinostat (Zolinza™, SAHA). RKO cells were treated with SAHA (1 µM, 24 hours). Consistent with previous studies [43,47], substantial changes in the global histone PTM patterns were observed (Supplementary Figure S8). Increases in the relative intensity of higher molecular weight peaks were observed for all the core histones relative to the histones from untreated cells. On histone H4, mass increases in units of 42 Da (3 m.e., corresponding to acetylation) were particularly evident. Notably, the observed changes in H2A and H2B were more substantial than those observed in previous studies using K562 and HT-29 cells [41,43]. These results suggest that SAHA treatment triggers extensive increases in *N*-acetylation of core histones, consistent with substantial HDAC inhibition.

### Detection of hypoxia-induced PTMs on intact core histones

**Figure 1.**
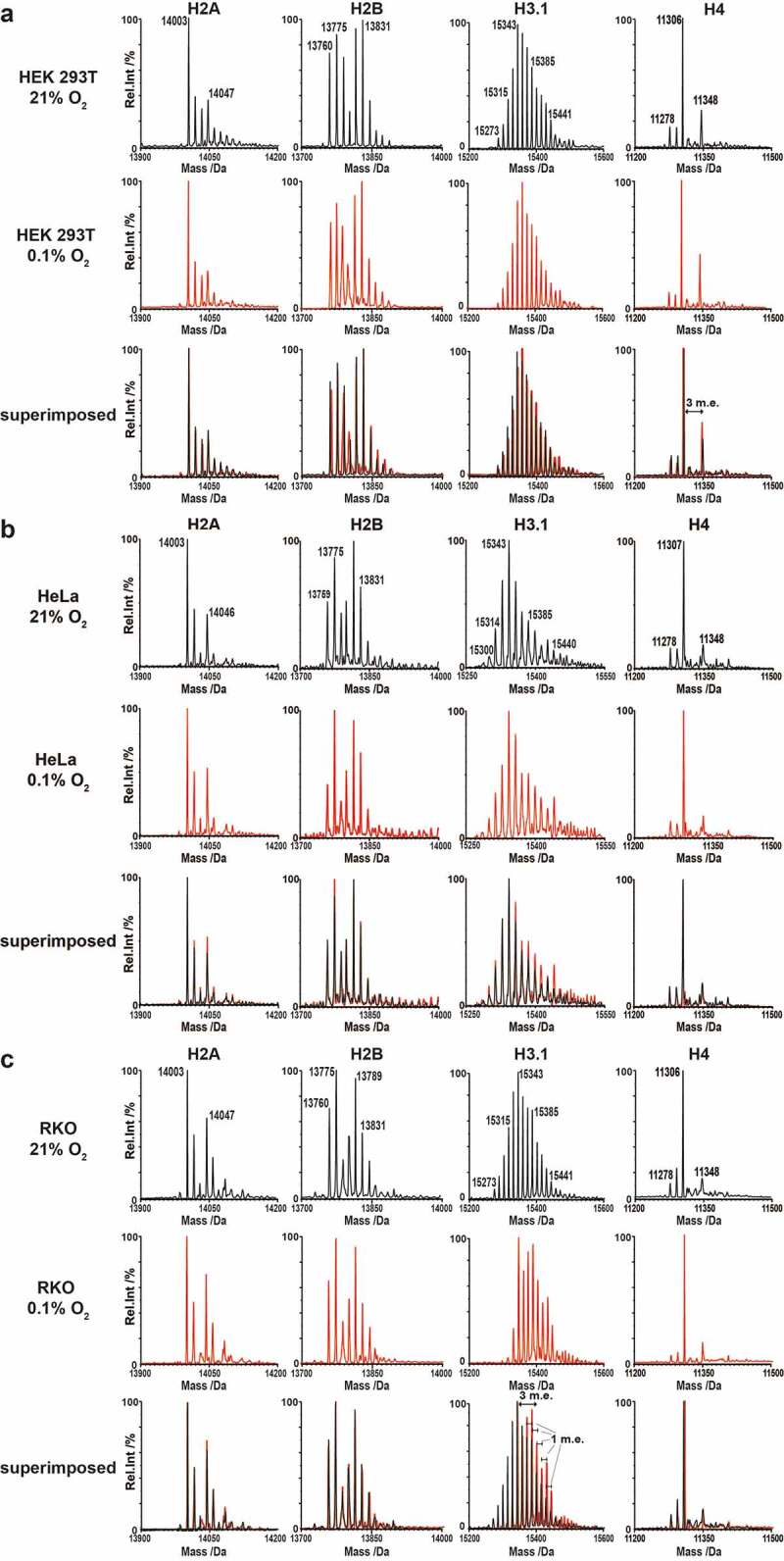
Deconvoluted MS profiles from LC-MS analysis of intact core histones under normoxia and hypoxia. Intact histone profiles from (a) HEK 293T, (b) HeLa and (c) RKO cells cultured for 24 hours under normoxia (21% O_2_) or severe hypoxia (0.1% O_2_). Coloured traces indicate the sample from control cells grown in normoxia (black) or cells treated under severe hypoxia for 24 hours (red)

Having validated our protocol, we investigated the effects of hypoxia and hypoxia mimetics on histone PTMs. Initially, we analysed histones from HEK 293T, HeLa and RKO cells grown under normoxia (21% O_2_) or severe hypoxia (0.1% O_2_) for 24 hours (Figure 1). The profiles of all histones shifted towards higher molecular weights, implying more highly modified states. In all cases, the most substantial changes in the mass profiles of intact histones in hypoxia were observed for histones H3 (shown for H3.1 in Figure 1) and H4. In the control cells, MS profiles of intact H3.1 displayed many peaks separated by 1 m.e.. Hypoxic treatment resulted in increases in the relative intensity of peaks at incremental shifts of 1 and 3 m.e. from the parent peak at 15273 Da in all cell lines, particularly for RKO cells (Figure 1, H3.1), with peaks visible at molecular weights as high as 15525 Da (+18 m.e., Figure 1(a,b)). The average molecular weight of H3.1 in RKO cells substantially increased (Figure 1(c)). The major peak observed for H4, in both normoxia and hypoxia in all cell types, remained around 11306 Da (+2 m.e.), which corresponds to the mass change associated with *N*-acetylated (*N*-Ac) H4 with an additional two methyl groups [40,48]. Changes in the intensities of other H4 peaks, relative to the 11306 Da peak, were observed between the hypoxic and normoxic samples. With the RKO cell samples, peaks corresponding to *N*-Ac H4 (11278 Da) and at 11292 Da (+1 m.e., mono-methylation) were decreased in severe hypoxia compared to normoxia (Figure 1(c), H4). The same trend was observed in HEK 293T cells, along with a concurrent increase of a peak at 11348 Da (+3 m.e.), likely corresponding to acetylation of the major H4 peak at 11278 Da (*N*-Ac H4). In severe hypoxia, no significant change in the PTM profiles were observed for H2A or H2B in all three cell lines, with the exception of H2A.X, where a substantial peak was observed at 15136 (+80) Da (Supplementary Figure S9); this corresponds to phosphorylation, a known marker of DNA damage [49]. Overall, the RKO cells exhibited more significant changes in their PTM profiles compared to the HEK 293T and HeLa cells.

### Effects of hypoxia mimetics on histone H3

**Figure 2.**
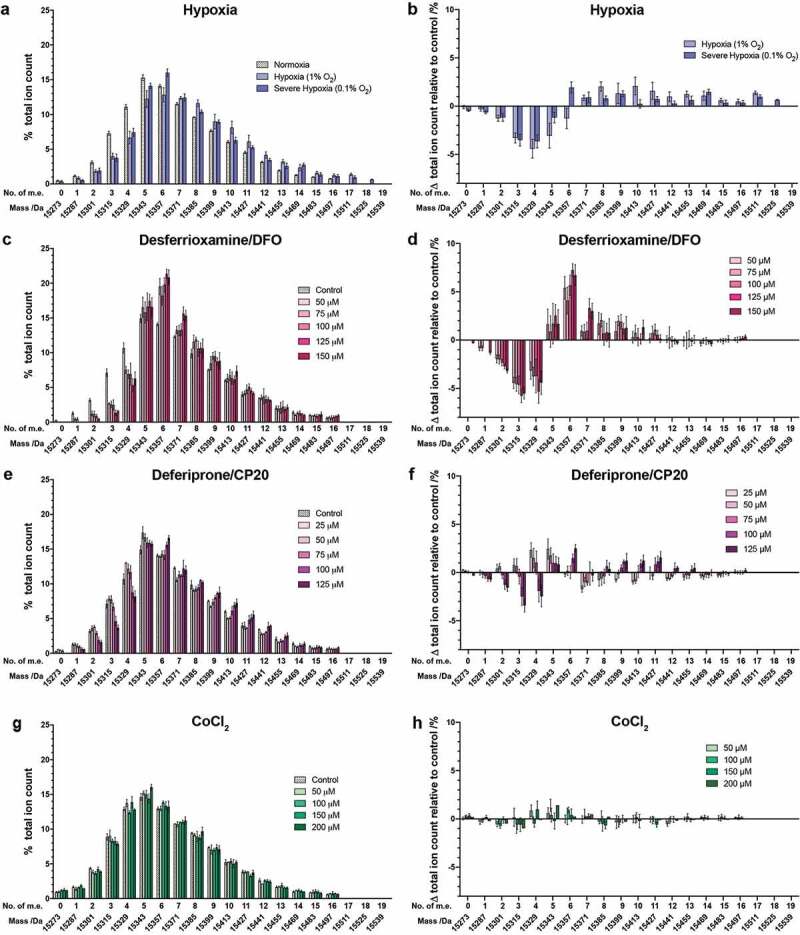
Histone H3.1 PTM profiles following treatment of cells with hypoxia or hypoxia mimetics. Histones were extracted from HEK 293T cells treated for 24 hours with: (a,b) normoxia (21% O_2_), hypoxia (1% O_2_), or severe hypoxia (0.1% O_2_); (c,d) 50, 75, 100, 125 and 150 μM DFO; (e,f) 25, 50, 75, 100 and 125 μM CP20; (g,h) 50, 100, 150 and 200 μM CoCl_2_. (a,c,e,g) The ion count for each mass spectral peak is expressed as a percentage of the total ion count. (b,d,f,h) The ion count for each peak as a percentage of the total ion count is expressed as a change relative to the peak intensity in control cells in each experiment. Data are expressed as mean ion counts ± SEM (n = 3). m.e.: number of methylation equivalents

We next compared the effect of hypoxia mimetics on the histones from HEK 293T cells with those we observed for hypoxia itself (Figure 2(a,b)). We focused our analyses on H3 and H4, because PTMs on H3 and H4 were most influenced by hypoxia (Figure 1). HEK 293T cells were treated with a range of concentrations of compounds over 24 hours before analysis. Very little reduction in cell viability was observed at any of the concentrations tested (Supplementary Figure S5). Dose-dependent effects of varying magnitudes were observed with each compound (Figure 2).

#### Hypoxia mimetics: iron chelators

We investigated the changes in histone PTM profiles observed on treatment of cells with the iron chelating drugs, DFO and CP20 (Supplementary Figure S1). Histone mass profiles were obtained from HEK 293T cells treated with five different concentrations of DFO (50, 75, 100, 125 and 150 μM) for 24 hours (Figure 2(c,d)), which revealed clear dose-dependent effects. In the untreated histone H3.1 mass profile, the most abundant peak was observed at 15343 Da (+5 m.e.), with a clear shift to 15357 Da in the treated cells (+6 m.e. – a change in mean ion intensity of ~7% between the untreated cells and those treated with 150 µM DFO, Figure 2(c,d)). The unmodified form (15273 Da) of H3.1 was not observed in any cell type under any of the DFO treatment concentrations. The apparent monomethylated form of H3.1 was not observed after treatment with more than 75 µM DFO either; the apparent dimethylated form was also not observed after treatment with 150 µM DFO. In general, the treatment with DFO induced large apparent changes in the abundance of species in the mass range of 15273 Da (unmodified) to 15,399 Da (+9 m.e.); however, no obvious abundance changes were observed in the species containing more than 11 m.e. (Figure 2(d)).

Analysis of HEK 293T cells treated with five different concentrations of CP20 (25, 50, 75, 100 and 125 μM) for 24 hours was carried out (Figure 2(e,f)). Dose-dependent effects on the histone PTM profile were again observed. At the highest two concentrations of CP20, unmodified H3.1 was not observed. With 125 μM CP20, the most abundant species was at 15357 Da (+6 m.e.), whereas with 0–100 μM CP20 the most abundant species was at 15343 Da (+5 m.e.) (Figure 2(e)). Overall, treatment with CP20 at 100–125 μM biased the intact H3.1 mass pattern towards more highly modified forms.

Overall, the magnitude of the observed changes differed significantly between compound treatments. In no cases did the iron chelators exactly replicate the effects of hypoxia on the distribution of histone PTMs.

#### Cobalt chloride (CoCl_2_)

Unlike the results for the iron chelators, treatment of HEK 293T cells with CoCl_2_ up to 200 μM resulted in only small apparent changes in the global PTM patterns on intact histone H3.1 (Figure 2(g,h,)). This observation suggests that, under the tested conditions, the changes in histone PTMs induced by Co(II) ions are less extensive than for the iron chelators, especially Exjade and DFO. Since all these treatments are reported to induce HIF-α at the tested concentrations, the combined results imply that changes in histone PTMs, including by the iron chelating drugs, are not directly mediated by HIF-α upregulation.

#### Oxygenase inhibitors: dimethyloxalylglycine (DMOG), IOX2 and FG4592

Given the ability of PHDs to regulate HIF levels, to investigate their (and other 2OG oxygenases) contribution to the observed histone PTM changes, we treated cells with the broad spectrum 2OG oxygenase inhibitor, DMOG (a prodrug form of N-oxalylglycine, a close 2OG isostere), and more specific PHD2 inhibitors, IOX2 and FG4592 (Supplementary Figure S1) [27,28,50].

**Figure 3.**
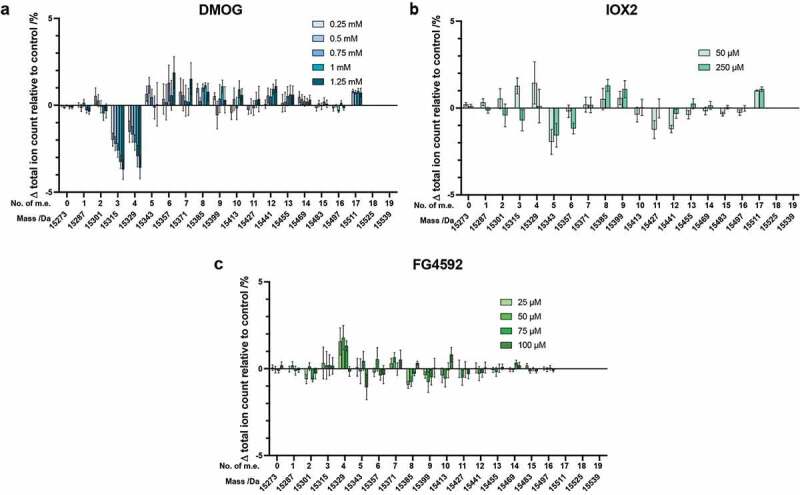
Histone H3.1 PTM profiles following treatment of cells with 2OG oxygenase inhibitors. Histones were extracted from HEK 293T cells treated for 24 hours with various concentrations of: (a) DMOG; (b) IOX2; (c) FG4592. The ion count for each peak as a percentage of the total ion count is expressed as a change relative to the control cells in each experiment. Data are means ± SEM (n = 3). m.e.: number of methylation equivalents

On treatment of cells with DMOG, discernible changes in the abundance of H3.1 PTMs were observed, with the most noticeable dose-dependent changes being for species containing +3 and +4 m.e. (Figure 3(a)). By contrast with the results for the iron chelating drugs and hypoxia, at even the highest concentration (1.25 mM), the unmodified form of H3.1 was observed, whilst the most highly modified H3.1 species was observed at 15511 Da (+17 m.e.) in cells treated with DMOG (0.5–1.25 mM), but not in the control cells. This observation is similar to that observed for cells grown under mild hypoxia (Figure 2(a,b)). As with hypoxia and the iron chelators, DMOG treatment slightly biased the overall H3 intact mass profile to more highly modified states in a dose-dependent manner.

Similarly to the results with Co(II) ions (Figure 2(g,h)), with IOX2 (50 and 250 µM) or FG4592 (25, 50, 75 and 100 µM), only very small changes were observed in the abundance of differently modified H3.1 peaks. All changes were much less prominent than for cells grown under hypoxia or treated with the iron chelating drugs or DMOG (Figure 2,3, Supplementary Figure S10). These results suggest that the majority of changes in H3.1 PTMs observed under hypoxia or with the other mimics tested are not directly caused by PHD-mediated HIF upregulation.

### Effect of hypoxia and hypoxia mimetics on histone H4

**Figure 4.**
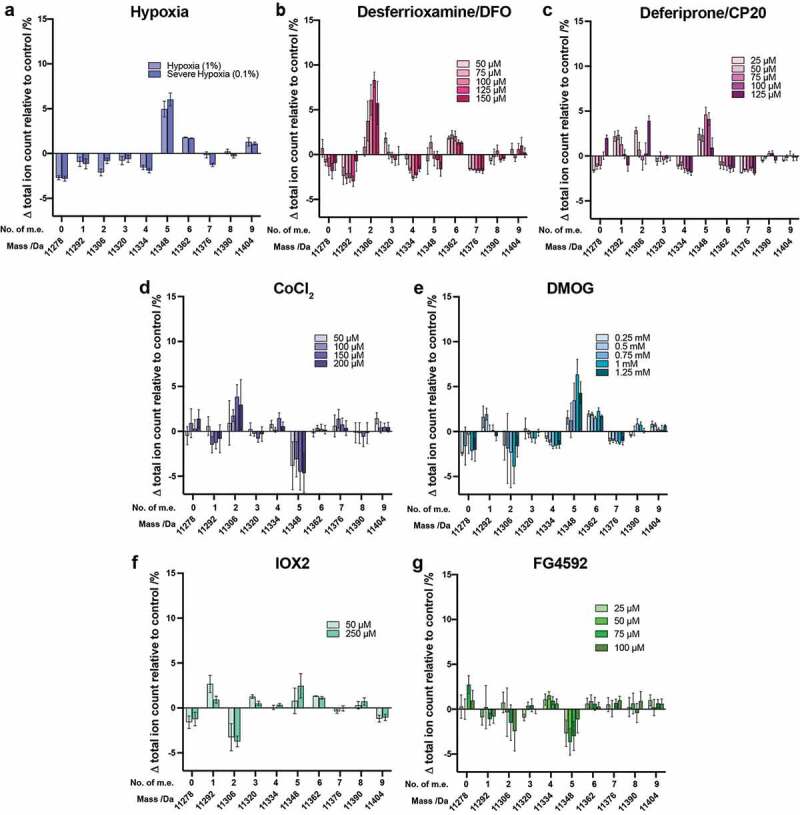
Analysis of histone H4 following treatment of cells with hypoxia, hypoxia mimetics or 2OG oxygenase inhibitors. Histones were extracted from HEK 293T cells treated for 24 hours with various concentrations of (a) hypoxia, (b) DFO, (c) CP20, (d) CoCl_2_, (e) DMOG, (f) IOX2 or (g) FG4592. The ion count for each peak as a percentage of the total ion count is expressed as a change relative to the control cells in each experiment. Data are means ± SEM (n = 3). No. of m.e.: number of methylation equivalents

Analysis of the intact H4 mass profile was also performed (Figure 4, Supplementary Figure S11). In hypoxia, the largest change was observed for the H4 species at 11348 Da (+5 m.e.), followed by the unmodified histone at 11278 Da (0 m.e.) (Figure 4(a)). Of note, the observed changes in the intact H4 mass profile were different between treatments with hypoxia and the three iron chelators (DFO and CP20). With the iron chelators, the abundance of the species at 11306 Da (+2 m.e.) increased most substantially. As for cells grown under hypoxia, treatment with CP20 also resulted in an increase in the +5 m.e. state (Figure 4(c)), but this was not observed for DFO (Figure 4(b)).

The overall results reveal the extent and direction of changes to H4 modifications caused by hypoxia and iron chelators differ substantially under the tested conditions. The change in pattern of the H4 mass profile on DMOG treatment was most similar to that observed in hypoxia (Figure 4(a,e)). As for H3.1, changes in H4 PTMs on treatment with IOX2 and FG4592 were fairly small (Figure 4(f,g), Supplementary Figure S11(f,g))

Based on previous reports it is likely that the species at 11306 Da, *N*-Ac H4 +2 m.e., correlates with the reported dimethylation of K20, and the species at 11348 Da (*N*-Ac H4 +5 m.e) correlates with *N*-Ac H4 +K20me2 plus a further acetylation (probably K16Ac)[51]. The results indicate that the hypoxia and DMOG treatments similarly manifest a higher population of this *N*-Ac H4 +5 m.e. peak, possibly due to inhibition of histone modifying enzymes (e.g. histone deacetylases, demethylases, acetyltransferases). Iron chelators and CoCl_2_ have comparable effects, causing a higher population of the species corresponding to *N*-Ac H4 +2 m.e. (11306 Da), again possibly due to enzyme inhibition. Interestingly, based on analysis of changes to both H3.1 and H4, treatment of cells with DMOG appears to be the best mimic of hypoxia on histone PTMs.

#### Time-dependent experiments of hypoxia and hypoxia mimetics

**Figure 5.**
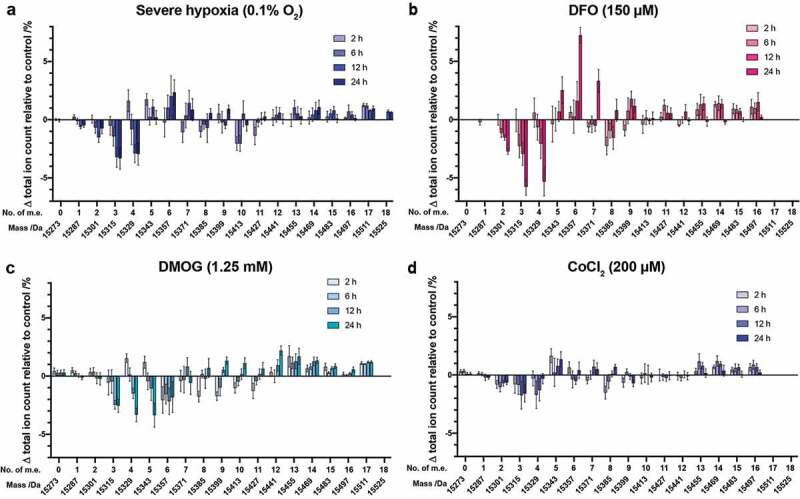
Changes in H3.1 PTM profiles over time following a range of hypoxic stresses. Histones were extracted from HEK 293T cells treated with (a) severe hypoxia (0.1% O_2_), (b) DFO (150 µM), (c) DMOG (1.25 mM), (d) CoCl_2_ (200 µM) for various times. The ion count for each peak as a percentage of the total ion count is expressed as a change relative to the control cells in each experiment. Data are means ± SEM (n = 3). No. of m.e: number of methylation equivalents

The time-dependent effects of hypoxia and hypoxia-mimetic agents on histones were then investigated. HEK 293T cells were treated with severe hypoxia (0.1% O_2_), or a single concentration of a hypoxia mimetic (150 μM DFO, 1.25 mM DMOG, or 200 μM CoCl_2_), for between 1 and 24 hours prior to analysis (Figure 5, Supplementary Figure S12). In all cases the extent of changes in the PTM profiles increased over the time period studied. In severe hypoxia, changes in PTM profiles became obvious after 6-h exposure, while the maximum effects were observed between 12 and 24 hours, suggesting cells might tolerate hypoxic stress for a short time, with more significant changes/adaptions appearing only over a longer stress exposure period (Figure 5(a)). Similarly, treatment of cells with DFO (150 μM) induced alterations in the intact H3.1 PTM profile after a 6-h exposure. Unlike the observations under severe hypoxia, however, dramatic changes continued to occur between the 12- and 24-h exposure (Figure 5(b)). With DMOG, changes in the H3.1 mass profile were detected more quickly, with evidence of significant changes after only a 2-h exposure; again the maximum effect was observed after a 24-h exposure (Figure 5(c)). Following CoCl_2_ treatment (200 μM), obvious changes were observed at all timepoints between 15315 Da (3 m.e.) and 15385 Da (8 m.e.) and above 15455 Da, while the other species changed only slightly in abundance over time (Figure 5(d)). These results indicate that the extent of time-dependent effects of hypoxia and hypoxia-mimetic agents (DFO, DMOG and CoCl_2_) on the global histone modification profiles also varies between stresses.

## Discussion

Whilst ‘bottom-up’ MS approaches (involving proteolytic fragmentation) can enable identification of histone PTMs at specific residues, their use for the analysis of multivalent, combinatorial PTMs and histone variants, is technically complicated and frequently ineffective. This is because digested peptides may not contain all the modified residues *in trans*, the rates and positions of digestion may differ with PTM patterns, peptide ionization efficiencies may vary, and because the same peptide sequences may coexist in different histone variants, which prevents their mapping back onto the full protein sequence [32,35,37,52]. Thus, complementary MS strategies, including analysis of intact histones, as employed in this study, have the potential to provide a useful perspective on global histone PTM profiles[53]. Limitations of intact protein analyses include lack of identification of specific PTMs (though these can sometimes be inferred) and their locations. A caveat to most strategies is that labile PTMs may be lost in the acidic histone extraction procedure.

We were interested to extend the intact protein protocol to profiling the global effect of cellular stresses on histone PTMs. Our protocol shows good reproducibility and coverage suggesting it is robust for detection of a range of histone isoforms and to profile their PTMs, consistent with the results from previous studies [39,54,55]. LC-MS analysis of intact histones may be of general use in selectivity studies on oxygenase and other enzyme inhibitors, including when histone modification is undesirable, i.e. one would look for compounds that do not cause changes in histone profiles.

The method was used to analyse global changes in histone PTM profiles resulting from hypoxia and commonly used hypoxia mimetics in human cell lines. Substantial changes of intact histone H3 and H4 PTMs were observed following the treatment of HEK 293T, HeLa and RKO cells with hypoxia. By contrast, much smaller changes in PTM profiles were observed on H2A and H2B. The exception to this was H2A.X; Phosphorylation at S139 of H2A.X (γ-H2AX) is a marker of DNA damage which can occur in severe hypoxia[49]. Interestingly, an increased level of (apparent) phosphorylation (+80 Da) was observed for H2A.X in RKO cells in severe hypoxia in our study, with a ratio of nearly 1:2 γ-H2AX:H2AX, whereas no increased level of phosphorylated H2A.X was noted in HEK 293T cells under the same hypoxic conditions (Supplementary Figure S9). Our findings suggest that different cell types may have varied H2A.X mediated responses to hypoxic stress.

Hypoxia is reported to induce changes in histone PTMs, both in local chromatin environments, but also, in some cases, globally [10,11,14,56]. Hypoxia induces context-dependent HIF target gene expression, but in general causes a reduction in transcription [10,56]. Hypoxia-mediated histone PTM changes (especially to H3) appear to be complex and may occur in a gene-specific manner. The global level of histone lysyl *N*^ε^-methylation is mediated by the activities of two functionally opposed enzyme families; the histone methyltransferases and demethylases (KDMs), the latter of which include both flavin-dependent enzymes and the 2OG-dependent JmjC KDMs. Increased activity of the methyltransferase G9a and the decreased activity of JmjC KDMs in 0.2% O_2_ have both been reported [14,15,57,58]. Some 2OG-dependent JmjC KDMs have also been identified to be transcriptionally induced by HIF in hypoxia [11,59,60]. As a result, in some cases, the induction of JmjC KDMs under hypoxic conditions may, at least in part, compensate for their reduced activity due to limiting oxygen [16,56]. Thus, the relationship between hypoxia and histone PTMs is complex.

Our results inform on the effects of hypoxia and hypoxia mimetics on histone PTM profiles. Two clinically used iron chelators (DFO and CP20), cobalt chloride, and 2OG oxygenase inhibitors, along with several different concentrations of oxygen were profiled. Overall, the results support previous work on hypoxia-/hypoxia-mimic-induced alterations in chromatin structure. All cellular stresses induced alterations to the global histone PTM profiles, but to different extents, as revealed by our LC-MS-based analyses of intact histones. Note, we have recently shown that Exjade also causes alterations in histone PTMs, at least in part through inhibition of the JmjC KDMs[30]. The dose- and time-dependencies of these effects were investigated to gain further insight into the effects of hypoxic/hypoxia-mimetic stresses. In most cases, H3.1 and H4 were found to shift to more highly modified states on hypoxia/compound treatment, likely substantially due to increased methylation. However, the results imply that, at least in cell culture, the extent of these changes varies widely between hypoxia and different hypoxia mimetics. Of the compounds tested, DMOG appeared to most closely mimic hypoxia. The precise reasons for the differences in histone PTMs observed are unknown, and likely reflect the complexity of epigenetic regulation.

Although care should be taken in assuming these cellular results apply *in vivo*, they imply that there are clear differences in the effects of physiological hypoxia and hypoxia mimetics. The magnitude of the observed changes from intact mass profiles differ considerably among hypoxia and chemical interventions. Given the complexity of the mammalian hypoxic response, these differences are not surprising, but they are nonetheless striking[1].

## Materials and methods

### Cell lines and cell culture

HEK 293T, HeLa, RKO and MCF-7 human cell lines were from the American Type Culture Collection (ATCC, Manassas, VA). Cells were cultivated in Dulbecco’s modiﬁed Eagle’s medium (DMEM, Lonza) supplemented with 10% foetal bovine serum (FBS, Invitrogen) and L-glutamine (Invitrogen) with/without penicillin (100 U/mL) and streptomycin (0.1 mg/mL). Cells were maintained in a Binder BD 53 incubator with 5% CO_2_ at 37°C. For experiments at reduced O_2_ concentrations, cells were cultured in a Baker Ruskinn Invivo2 400 hypoxic chamber with 5% CO_2_ at 37°C.

### Trypan blue exclusion assay

Cells were treated with variable levels of hypoxia or concentrations of compounds for 24 hours. Trypan blue exclusion assays were then carried out following literature procedures to assess changes in membrane integrity[46]. Briefly, cells were resuspended to a density of 5 × 10^5^ cells/mL. 10–20 µL cell suspension was mixed with 0.4% trypan blue and incubated for 3 min at room temperature. A drop of this cell mixture was placed onto a haemocytometer and stained (dead cells) and unstained (viable cells) counted separately using a binocular microscope. Individual counts were averaged over four 0.1 mm^3^ squares. Three biological replicates were carried out for each condition.

### Hypoxia and hypoxia mimetic experiments

Cells were incubated in optimal condition (95% air and 5% CO_2_, 37°C) and grown to approximately 75% confluence before exposure to different O_2_ concentrations (21%, 5%, 1% and 0.1% O_2_) at 37°C for 24 h. Cells were also exposed to 0.1% O_2_ and were harvested at 2, 6, 12, and 24-h timepoints after 0.1% O_2_ exposure to test the time-dependent effect of hypoxia. In experiments with hypoxia mimetics, varying concentrations of DFO (25–150 μM, Sigma-Aldrich), CP20 (25–125 μM, Sigma-Aldrich), DMOG (250–1250 μM, Sigma-Aldrich) and CoCl_2_ (50–200 μM, Sigma-Aldrich) were used to treat cells at approximate 75% confluence (95% air and 5% CO_2_ incubator at 37°C, 24 hours). In addition, for time-dependent experiments, DFO (150 μM), DMOG (1250 μM) and CoCl_2_ (200 μM) were used to treat cells before harvesting at 2, 6, 12, and 24-h post compound treatment. Compounds (dissolved in doubly distilled water or DMSO) were added directly into the cell culture medium to the desired final concentrations.

### Preparation of histones for LC-MS analysis

Histones were extracted from cells and prepared for LC-MS analysis based on literature procedures [44,54]. Prepared cell pellets (HEK 293T, HeLa, MCF-7, or RKO cells) were resuspended in ice-cold hypotonic lysis buffer (10 mM Tris-Cl pH 8.0, 1 mM KCl, 1.5 mM MgCl_2_, 1 mM dithiothreitol and 1 mM phenylmethanesulfonylfluoride, supplemented with 1x protease and phosphatase inhibitors (Thermo Fisher Scientific) then incubated on a rotator at 4°C for 30 minutes. The nuclei were pelleted by centrifugation (10,000 x g, 4°C, 10 min), and the supernatant discarded. The pellets were resuspended in 400 μL 0.4 N ice-cold HCl. Samples were centrifuged (16,000 x g, 4°C, 10 min) and the supernatant containing histones transferred into an Eppendorf tube. Following acid extraction, ~400 μL supernatant was added to a 15 mL falcon tube with 4 mL acetone and placed at −20°C overnight for precipitation. The sample was then centrifuged (2,500 x g, 4°C, 10 min), the supernatant discarded, and the pellet transferred into the fresh 1.5 mL Eppendorf tube. Three washes with ice-cold acetone were carried out by centrifugation (16,000 x g, 4°C, 5 min) and the pellet dried at room temperature. The appropriate volume of 0.1% (v/v) aqueous formic acid (typically 100 μL) was added to dissolve the final pellet and the solution was stored at −20°C.

### LC-MS histone analyses

Samples of histones, extracted as described above, were separated by reversed phase ultra-performance liquid chromatography (RP-UPLC) and analysed by electrospray ionization time-of-flight mass spectrometry (ESI-TOF MS) using a Waters Acquity UPLC system connected directly to a Waters LCT ESI-TOF mass spectrometer. UPLC separation was carried out at a flow rate of 0.25 mL/min on a Waters BEH C4 reversed phase column (2.1 x 150 mm, 1.7 μm particle size, 300 Å pore size) at 40°C. The column was equilibrated with solvent A (0.5% (v/v) formic acid in doubly distilled water) and solvent B (0.5% (v/v) formic acid in acetonitrile). 5 μL of histone sample was injected into the column and histones were eluted using a stepped gradient from solvent A to solvent B (Supplementary Figure S3).

The LCT-TOF MS (Waters Corp, Manchester, UK) parameter settings were as follows: polarity mode: ES+; capillary voltage: 3,000 V; sample cone voltage: 35 V; extraction cone voltage: 2.5 V; desolvation temperature: 250°C; cone gas flow rate: 10 L/hour; desolvation gas flow (N_2_): 500 L/hour. The acquisition mass range was 200 to 2000 *m/z* using MassLynx 4.1 instrument control software (Waters Corp, Manchester, UK). Mass spectral charge state envelopes were deconvoluted to determine histone molecular weights using Maxent 1 (MassLynx application manager that uses an entropy-based deconvolution algorithm to estimate molecular mass from multiple-charge state protein mass spectra) [61,62], using with mass accuracy 70 ppm with continuum mode data acquired at a rate of 1 spectrum/s. Masses were validated by manual analysis. Leu-Enkephalin was used as a lock spray reagent for calibration of the mass spectrometer at the monoisotopic mass of 556.277 [M + H]^+^.

Deconvoluted MS profiles are presented as the intensity of each species normalized relative to the intensity of the most abundant species. To allow comparison of species from different samples and treatment conditions, MS profiles were also transformed such that the ion count of each species is expressed as a percentage of the total ion count.

Results are presented as the means ± SD or SEM of three independent replicates.

## Disclosure of potential conflicts of interest

The authors declare no potential conflicts of interest.

## Supplementary Material

Supplemental Material

## References

[1] Kaelin WG Jr., Ratcliffe PJ. Oxygen sensing by metazoans: the central role of the HIF hydroxylase pathway. Mol Cell. 2008;30:393–402. 18498744 10.1016/j.molcel.2008.04.009

[2] Semenza GL. Hypoxia-inducible factor 1: oxygen homeostasis and disease pathophysiology. Trends Mol Med. 2001;7:345–350. 11516994 10.1016/s1471-4914(01)02090-1

[3] Wang GL, Semenza GL. General involvement of hypoxia-inducible factor 1 in transcriptional response to hypoxia. Proc Natl Acad Sci. 1993;90:4304 LP– 4308. 8387214 10.1073/pnas.90.9.4304 PMC46495

[4] Jaakkola P, Mole DR, Tian Y-M, et al. Targeting of HIF-α to the von hippel-lindau ubiquitylation complex by O2-regulated prolyl hydroxylation. Science. 2001;292:468–472. 11292861 10.1126/science.1059796

[5] Epstein ACR, Gleadle JM, McNeill LA, et al. C. elegans EGL-9 and mammalian homologs define a family of dioxygenases that regulate HIF by prolyl hydroxylation. Cell. 2001;107:43–54. 11595184 10.1016/s0092-8674(01)00507-4

[6] Bruick RK, McKnight SL. A Conserved Family of Prolyl-4-Hydroxylases That Modify HIF. Science. 2001;294:1337–1340. 11598268 10.1126/science.1066373

[7] Batie M, Del Peso L, Rocha S. Hypoxia and chromatin: A focus on transcriptional repression mechanisms. Biomedicines. 2018;6:1–19. 10.3390/biomedicines6020047 PMC6027312 29690561

[8] Perez-Perri JI, Acevedo JM, Wappner P. Epigenetics: new questions on the response to hypoxia. Int J Mol Sci. 2011;12:4705–4721. 21845106 10.3390/ijms12074705 PMC3155379

[9] Choudhry H, Harris AL. Advances in hypoxia-inducible factor biology. Cell Metab. 2017;27:281–298. 29129785 10.1016/j.cmet.2017.10.005

[10] Johnson AB, Denko N, Barton MC. Hypoxia induces a novel signature of chromatin modifications and global repression of transcription. Mutat Res. 2008;640:174–179. 18294659 10.1016/j.mrfmmm.2008.01.001 PMC2346607

[11] Pollard PJ, Loenarz C, Mole D, et al. Regulation of Jumonji-domain-containing histone demethylases by hypoxia-inducible factor (HIF)-1a. Biochem J. 2008;416:387–394. 18713068 10.1042/BJ20081238

[12] Hancock RL, Dunne K, Walport LJ, et al. Epigenetic regulation by histone demethylases in hypoxia. Epigenomics. 2015;7:791–811. 25832587 10.2217/epi.15.24

[13] Tausendschön M, Dehne N, Brüne B. Hypoxia causes epigenetic gene regulation in macrophages by attenuating Jumonji histone demethylase activity. Cytokine. 2011;53:256–262. 21131212 10.1016/j.cyto.2010.11.002

[14] Prickaerts P, Adriaens ME, Beucken TVD, et al. Hypoxia increases genome-wide bivalent epigenetic marking by specific gain of H3K27me3. Epigenet Chromatin. 2016;9:1–19. 10.1186/s13072-016-0086-0 PMC5080723 27800026

[15] Hancock RL, Masson N, Dunne K, et al. The activity of JmjC histone lysine demethylase KDM4A is highly sensitive to oxygen concentrations. ACS Chem Biol. 2017;12:1011–1019. 28051298 10.1021/acschembio.6b00958 PMC5404277

[16] Batie M, Frost J, Frost M, et al. Hypoxia induces rapid changes to histone methylation and reprograms chromatin. Science. 2019;363:1222–1226. 30872526 10.1126/science.aau5870

[17] Klose RJ, Kallin EM, Zhang Y. JmjC-domain-containing proteins and histone demethylation. Nat Rev Genet. 2006;7:715–727. 16983801 10.1038/nrg1945

[18] Walport LJ, Hopkinson RJ, Schofield CJ. Mechanisms of human histone and nucleic acid demethylases. Curr Opin Chem Biol. 2012;16:525–534. 23063108 10.1016/j.cbpa.2012.09.015

[19] Knowles HJ, Raval RR, Harris AL, et al. Effect of Ascorbate on the Activity of Hypoxia-inducible Factor in Cancer Cells. Cancer Res. 2003;63:1764 LP– 1768. 12702559

[20] Cho EA, Song HK, Lee S-H, et al. Differential in vitro and cellular effects of iron chelators for hypoxia inducible factor hydroxylases. J Cell Biochem. 2012;114:864–873. 10.1002/jcb.24423 23097160

[21] Tian Y-M, Yeoh KK, Lee MK, et al. Differential sensitivity of hypoxia inducible factor hydroxylation sites to hypoxia and hydroxylase inhibitors. J Biol Chem. 2011;286:13041–13051. 21335549 10.1074/jbc.M110.211110 PMC3075650

[22] Nurchi VM, Crisponi G, Lachowicz JI, et al. Chemical features of in use and in progress chelators for iron overload. J Trace Elem Med Biol. 2016;38:10–18. 27365273 10.1016/j.jtemb.2016.05.010

[23] Moukalled NM, Bou-Fakhredin R, Taher AT. Deferasirox: over a decade of experience in thalassemia. Mediterr J Hematol Infect Dis. 2018;10:1–13. 10.4084/MJHID.2018.066 PMC6223547 30416698

[24] Rose NR, McDonough MA, King ONF, et al. Inhibition of 2-oxoglutarate dependent oxygenases. Chem Soc Rev. 2011;40:4364–4397. 21390379 10.1039/c0cs00203h

[25] Bush JT, Chan MC, Mohammed S, et al. Quantitative MS-based proteomics comparing the MCF-7 cellular response to hypoxia and a 2-oxoglutarate analogue. Chembiochem. 2020;21:1647–1655. 10.1002/cbic.201900719 PMC7317498 31919953

[26] Maxwell P, Salnikow K. HIF-1: an oxygen and metal responsive transcription factor. Cancer Biol Ther. 2004;3:29–35. 14726713 10.4161/cbt.3.1.547

[27] Chowdhury R, Candela-Lena JI, Chan MC, et al. Selective small molecule probes for the hypoxia inducible factor (HIF) prolyl hydroxylases. ACS Chem Biol. 2013;8:1488–1496. 23683440 10.1021/cb400088q

[28] Becker K, Saad M. A new approach to the management of anemia in CKD patients: a review on roxadustat. Adv Ther. 2017;34:848–853. 28290095 10.1007/s12325-017-0508-9

[29] Pogribny IP, Tryndyak VP, Pogribna M, et al. Modulation of intracellular iron metabolism by iron chelation affects chromatin remodeling proteins and corresponding epigenetic modifications in breast cancer cells and increases their sensitivity to chemotherapeutic agents. Int J Oncol. 2013;42:1822–1832. 23483119 10.3892/ijo.2013.1855

[30] Roatsch M, Hoffmann I, Abboud MI, et al. The clinically used iron chelator deferasirox is an inhibitor of epigenetic JumonjiC domain-containing histone demethylases. ACS Chem Biol. 2019;14:1737–1750. 31287655 10.1021/acschembio.9b00289

[31] Sidoli S, Garcia BA. Properly reading the histone code by MS-based proteomics. Proteomics. 2015;15:2901–2902. 26223514 10.1002/pmic.201500298

[32] Sidoli S, Cheng L, Jensen ON. Proteomics in chromatin biology and epigenetics: elucidation of post-translational modifications of histone proteins by mass spectrometry. J Proteomics. 2012;75:3419–3433. 22234360 10.1016/j.jprot.2011.12.029

[33] Eberl HC, Mann M, Vermeulen M. Quantitative proteomics for epigenetics. Chembiochem. 2011;12:224–234. 21243711 10.1002/cbic.201000429

[34] Thomas CE, Kelleher NL, Mizzen CA. Mass spectrometric characterization of human histone H3: a bird’s eye view. J Proteome Res. 2006;5: 240–247. 16457588 10.1021/pr050266a

[35] Moradian A, Kalli A, Sweredoski MJ, et al. The top-down, middle-down, and bottom-up mass spectrometry approaches for characterization of histone variants and their post-translational modifications. Proteomics. 2014;14:489–497. 24339419 10.1002/pmic.201300256

[36] Aebersold R, Mann M. Mass spectrometry-based proteomics. Nature. 2003;422:198–207. 12634793 10.1038/nature01511

[37] Sidoli S, Lopes M, Lund PJ, et al. A mass spectrometry-based assay using metabolic labeling to rapidly monitor chromatin accessibility of modified histone proteins. Sci Rep. 2019;9:1–15. 31541121 10.1038/s41598-019-49894-4 PMC6754405

[38] Naldi M, Andrisano V, Fiori J, et al. Histone proteins determined in a human colon cancer by high-performance liquid chromatography and mass spectrometry. J Chromatogr A. 2006;1129:73–81. 16887128 10.1016/j.chroma.2006.06.100

[39] Su X, Jacob NK, Amunugama R, et al. Liquid chromatography mass spectrometry profiling of histones. J Chromatogr B Anal Technol Biomed Life Sci. 2007;850:440–454. 10.1016/j.jchromb.2006.12.037 PMC2694509 17254850

[40] Contrepois K, Ezan E, Mann C, et al. Ultra-high performance liquid chromatography-mass spectrometry for the fast profiling of histone post-translational modifications. J Proteome Res. 2010;9:5501–5509. 20707390 10.1021/pr100497a

[41] Drogaris P, Villeneuve V, Pomiès C, et al. Histone deacetylase inhibitors globally enhance h3/h4 tail acetylation without affecting h3 lysine 56 acetylation. Sci Rep. 2012;2:220. 22355734 10.1038/srep00220 PMC3256565

[42] Li M, Jiang L, Kelleher NL. Global histone profiling by LC-FTMS after inhibition and knockdown of deacetylases in human cells. J Chromatogr B Anal Technol Biomed Life Sci. 2009;877:3885–3892. 10.1016/j.jchromb.2009.09.042 PMC2783324 19828382

[43] Naldi M, Calonghi N, Masotti L, et al. Histone post-translational modifications by HPLC-ESI-MS after HT29 cell treatment with histone deacetylase inhibitors. Proteomics. 2009;9:5437–5445. 19834889 10.1002/pmic.200800866

[44] Shechter D, Dormann HL, Allis CD, et al. Extraction, purification and analysis of histones. Nat Protoc. 2007;2:1445–1457. 17545981 10.1038/nprot.2007.202

[45] Khare SP, Habib F, Sharma R, et al. HIstome - A relational knowledgebase of human histone proteins and histone modifying enzymes. Nucleic Acids Res. 2012;40:337–342. 10.1093/nar/gkr1125 PMC3245077 22140112

[46] Strober W. Trypan Blue Exclusion Test of Cell Viability. Curr Protoc Immunol. 2001;21:A.3B.1-A.3B.2. 10.1002/0471142735.ima03bs21 18432654

[47] Marks PA, Breslow R. Dimethyl sulfoxide to vorinostat: development of this histone deacetylase inhibitor as an anticancer drug. Nat Biotechnol. 2007;25:84–90. 17211407 10.1038/nbt1272

[48] Hole K, Van Damme P, Dalva M, et al. The human N-Alpha-acetyltransferase 40 (hNaa40p/hNatD) is conserved from yeast and N-terminally acetylates histones H2A and H4. PLoS One. 2011;6:1–11. 10.1371/journal.pone.0024713 PMC3174195 21935442

[49] Rogakou EP, Pilch DR, Orr AH, et al. DNA double-stranded breaks induce histone H2AX phosphorylation on serine 139. J Biol Chem. 1998;273:5858–5868. 9488723 10.1074/jbc.273.10.5858

[50] Rose NR, Ng SS, Mecinoić J, et al. Inhibitor scaffolds for 2-oxoglutarate-dependent histone lysine demethylases. J Med Chem. 2008;51:7053–7056. 18942826 10.1021/jm800936s

[51] Pesavento JJ, Mizzen CA, Kelleher NL. Quantitative analysis of modified proteins and their positional isomers by tandem mass spectrometry: human histone H4. Anal Chem. 2006;78:4271–4280. 16808433 10.1021/ac0600050

[52] Noberini R, Sigismondo G, Bonaldi T. The contribution of mass spectrometry-based proteomics to understanding epigenetics. Epigenomics. 2016;8:429–445. 26606673 10.2217/epi.15.108

[53] Molden RC, Garcia BA. Middle-down and top-down mass spectrometric analysis of co-occurring histone modifications. Curr Protoc Protein Sci. 2014;77:23.7.1–23.7.28. 10.1002/0471140864.ps2307s77 PMC4160057 25081742

[54] You J, Wang L, Saji M, et al. High-sensitivity TFA-free LC-MS for profiling histones. Proteomics. 2011;11:3326–3334. 21751347 10.1002/pmic.201000445 PMC3517135

[55] Galasinski SC, Resing KA, Ahn NG. Protein mass analysis of histones. Methods. 2003;31:3–11. 12893168 10.1016/s1046-2023(03)00082-3

[56] Xia X, Lemieux ME, Li W, et al. Integrative analysis of HIF binding and transactivation reveals its role in maintaining histone methylation homeostasis. Proc Natl Acad Sci. 2009;106:4260–4265. 19255431 10.1073/pnas.0810067106 PMC2657396

[57] Chen H, Yan Y, Davidson TL, et al. Hypoxic stress induces dimethylated histone H3 lysine 9 through histone methyltransferase G9a in mammalian cells. Cancer Res. 2006;66:9009–9016. 16982742 10.1158/0008-5472.CAN-06-0101

[58] Chakraborty AA, Laukka T, Myllykoski M, et al. Histone demethylase KDM6A directly senses oxygen to control chromatin and cell fate. Science. 2019;363:1217–1222. 30872525 10.1126/science.aaw1026 PMC7336390

[59] Yang J, Ledaki I, Turley H, et al. Role of hypoxia-inducible factors in epigenetic regulation via histone demethylases. Ann N Y Acad Sci. 2009;1177:185–197. 19845621 10.1111/j.1749-6632.2009.05027.x

[60] Wellmann S, Bettkober M, Zelmer A, et al. Hypoxia upregulates the histone demethylase JMJD1A via HIF-1. Biochem Biophys Res Commun. 2008;372:892–897. 18538129 10.1016/j.bbrc.2008.05.150

[61] Ferrige AG, Seddon MJ, Jarvis S, et al. Maximum entropy deconvolution in electrospray mass spectrometry. Rapid Commun Mass Spectrom. 1991;5:374–377.

[62] Ferrige AG, Seddon MJ, Green BN, et al. Disentangling electrospray spectra with maximum entropy. Rapid Commun Mass Spectrom. 1992;6:707–711.

