# Supplementary figures and images for: Hypoxia and hypoxia mimetics differentially modulate histone post-translational modifications
Source: Epigenetics. 2020 Jul 1;16(1):14–27. doi: 10.1080/15592294.2020.1786305 (PMC7889154; doi:10.1080/15592294.2020.1786305)

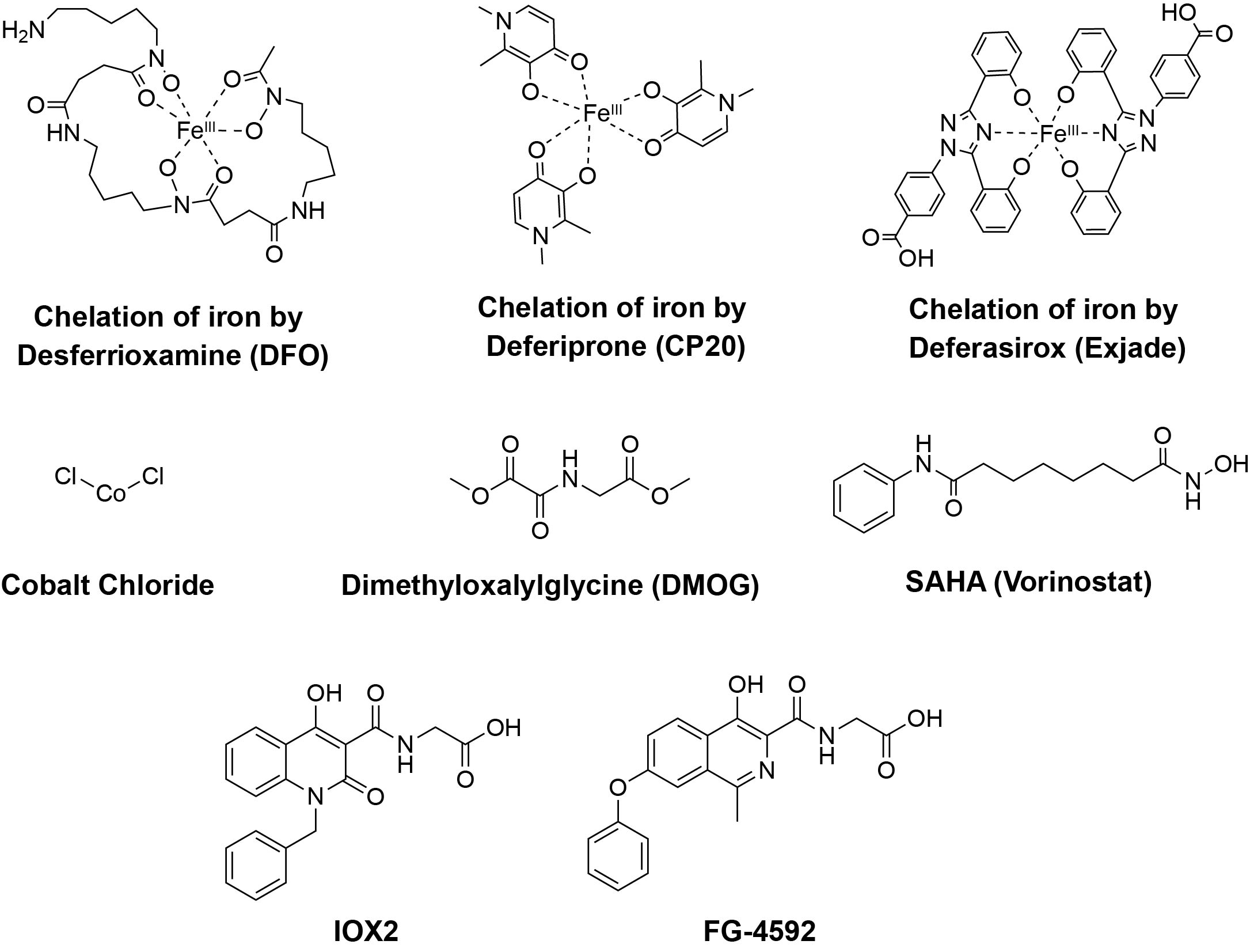

Supplement: Supplemental Material [file KEPI_A_1786305_SM8513.zip › Figure S1.jpg]

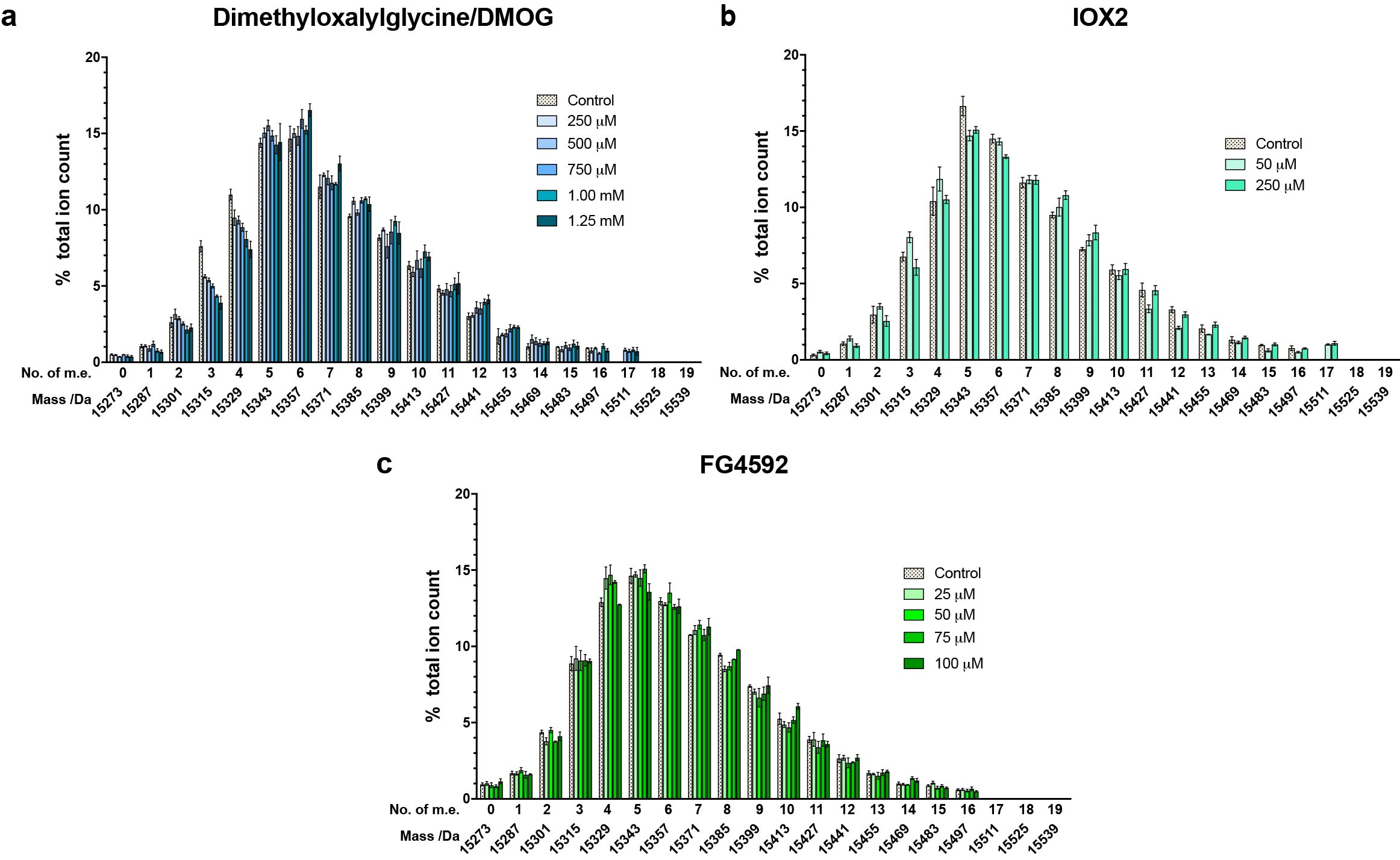

Supplement: Supplemental Material [file KEPI_A_1786305_SM8513.zip › Figure S10.jpg]

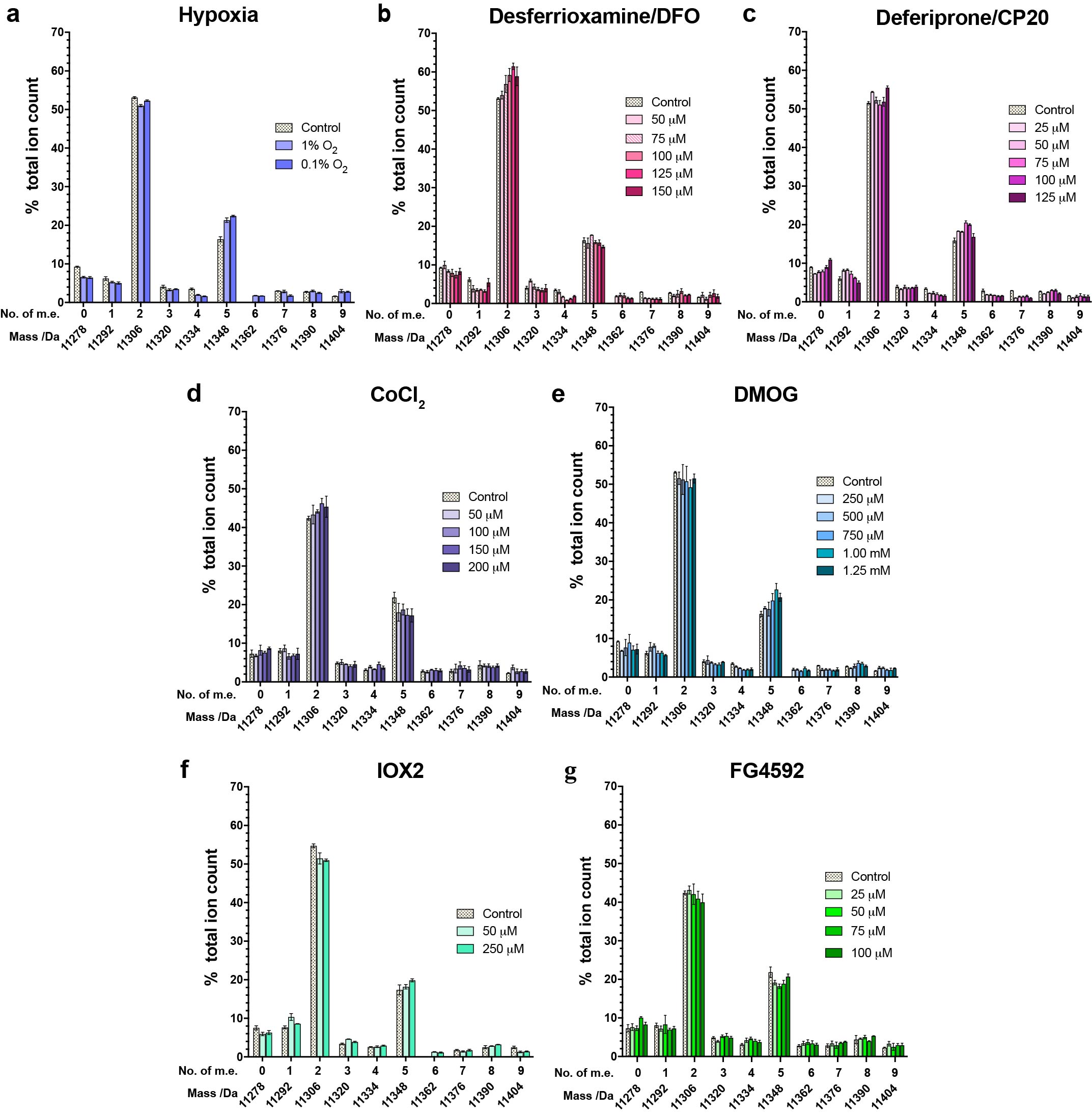

Supplement: Supplemental Material [file KEPI_A_1786305_SM8513.zip › Figure S11.jpg]

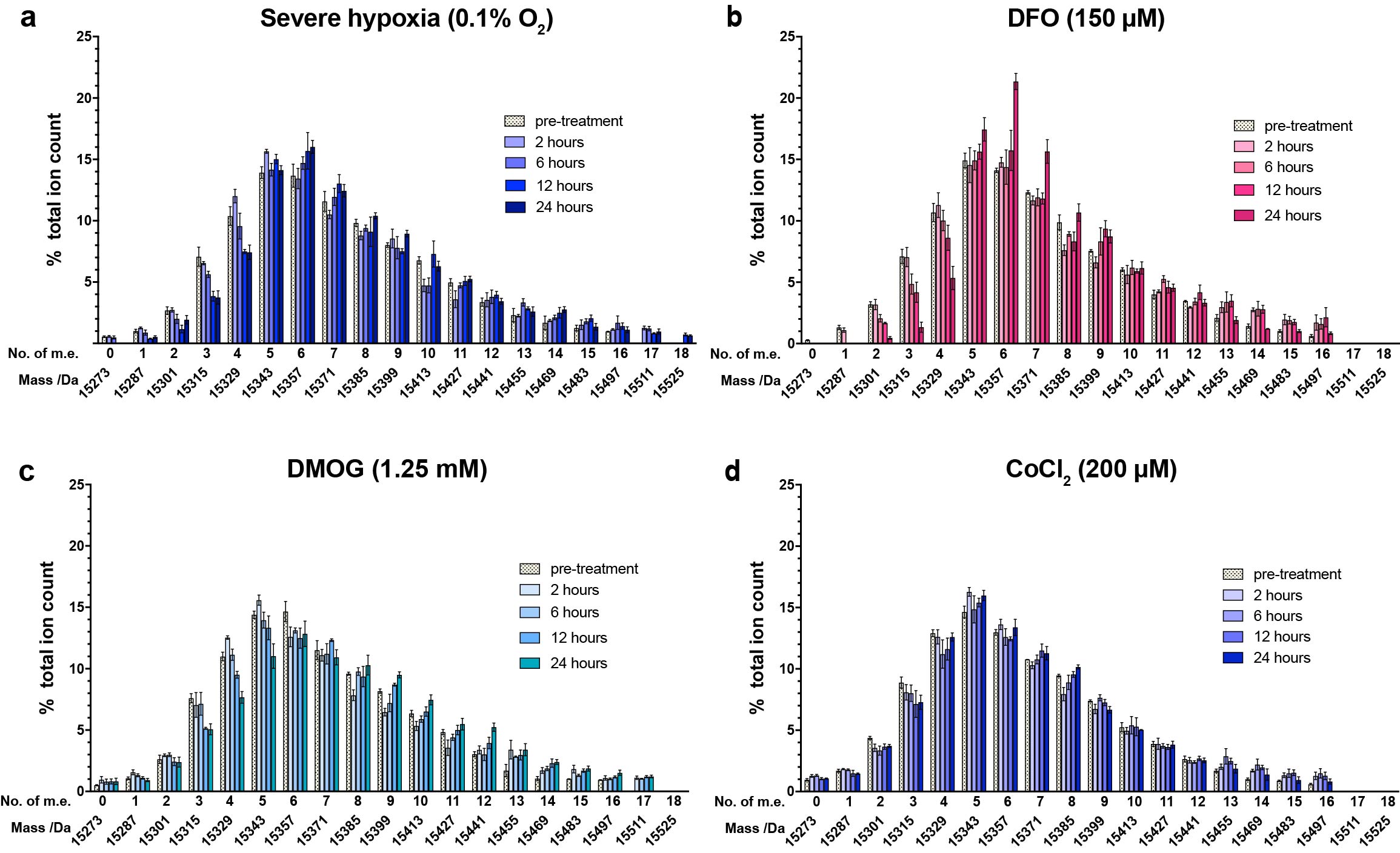

Supplement: Supplemental Material [file KEPI_A_1786305_SM8513.zip › Figure S12.jpg]

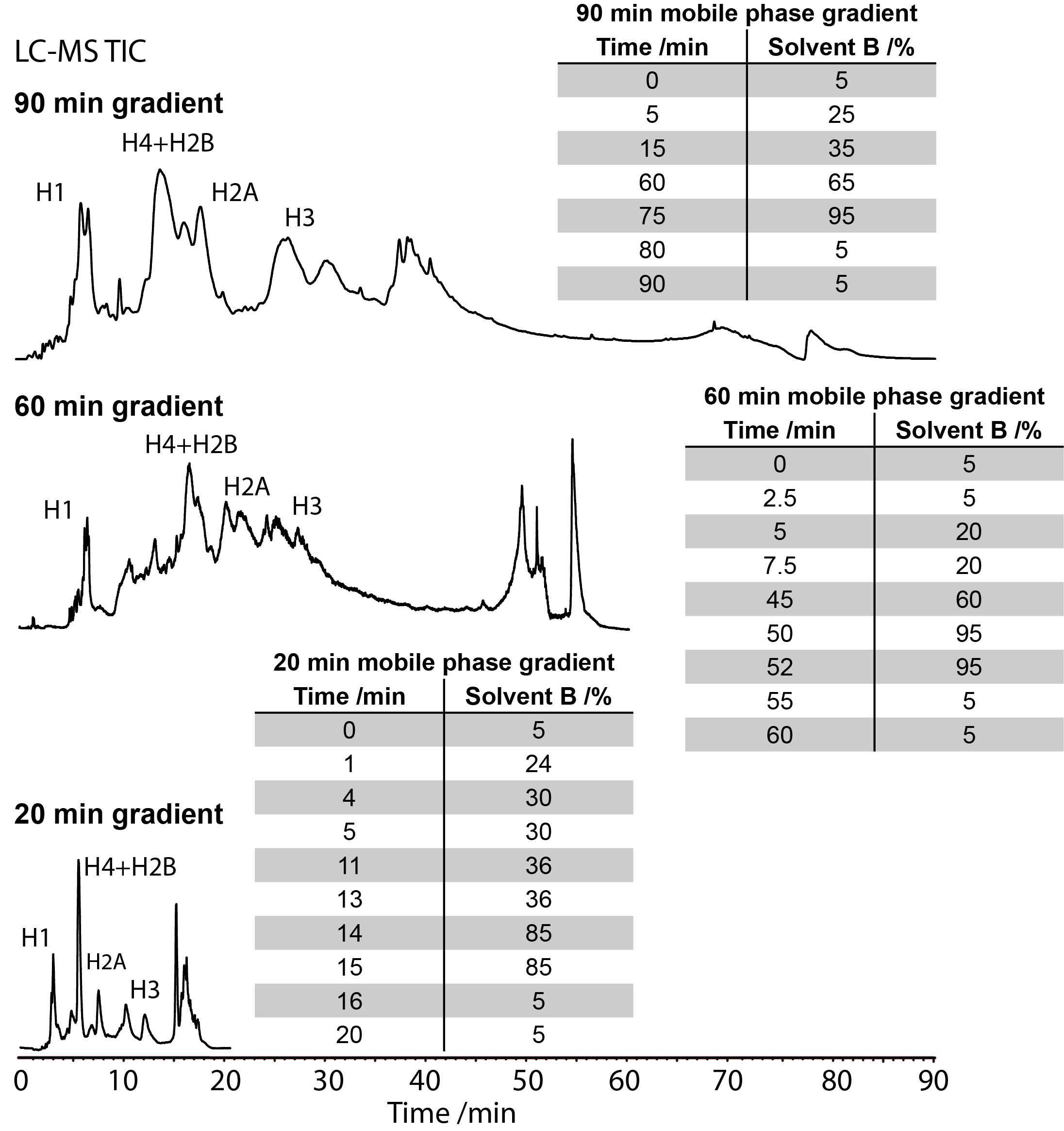

Supplement: Supplemental Material [file KEPI_A_1786305_SM8513.zip › Figure S2.jpg]

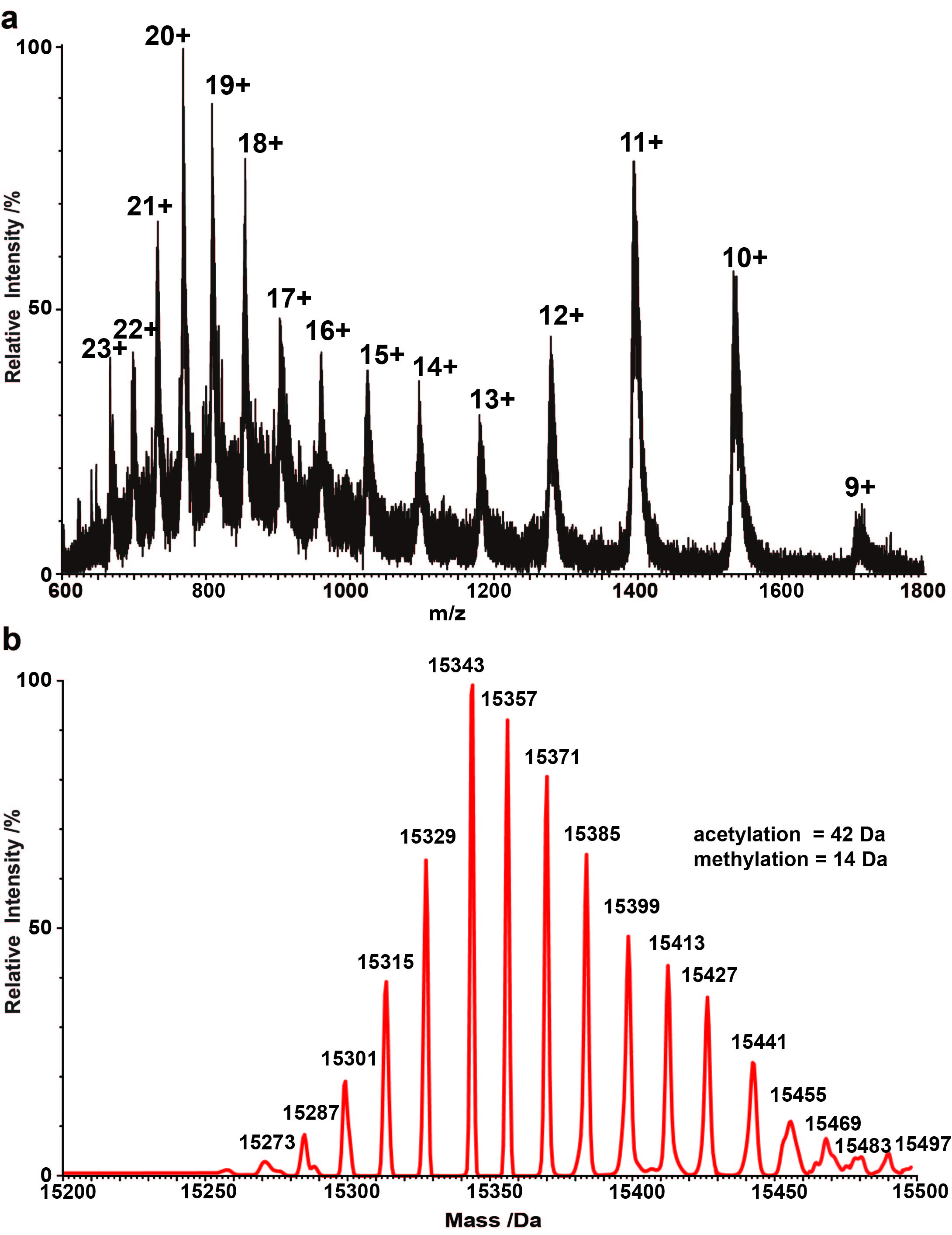

Supplement: Supplemental Material [file KEPI_A_1786305_SM8513.zip › Figure S3.jpg]

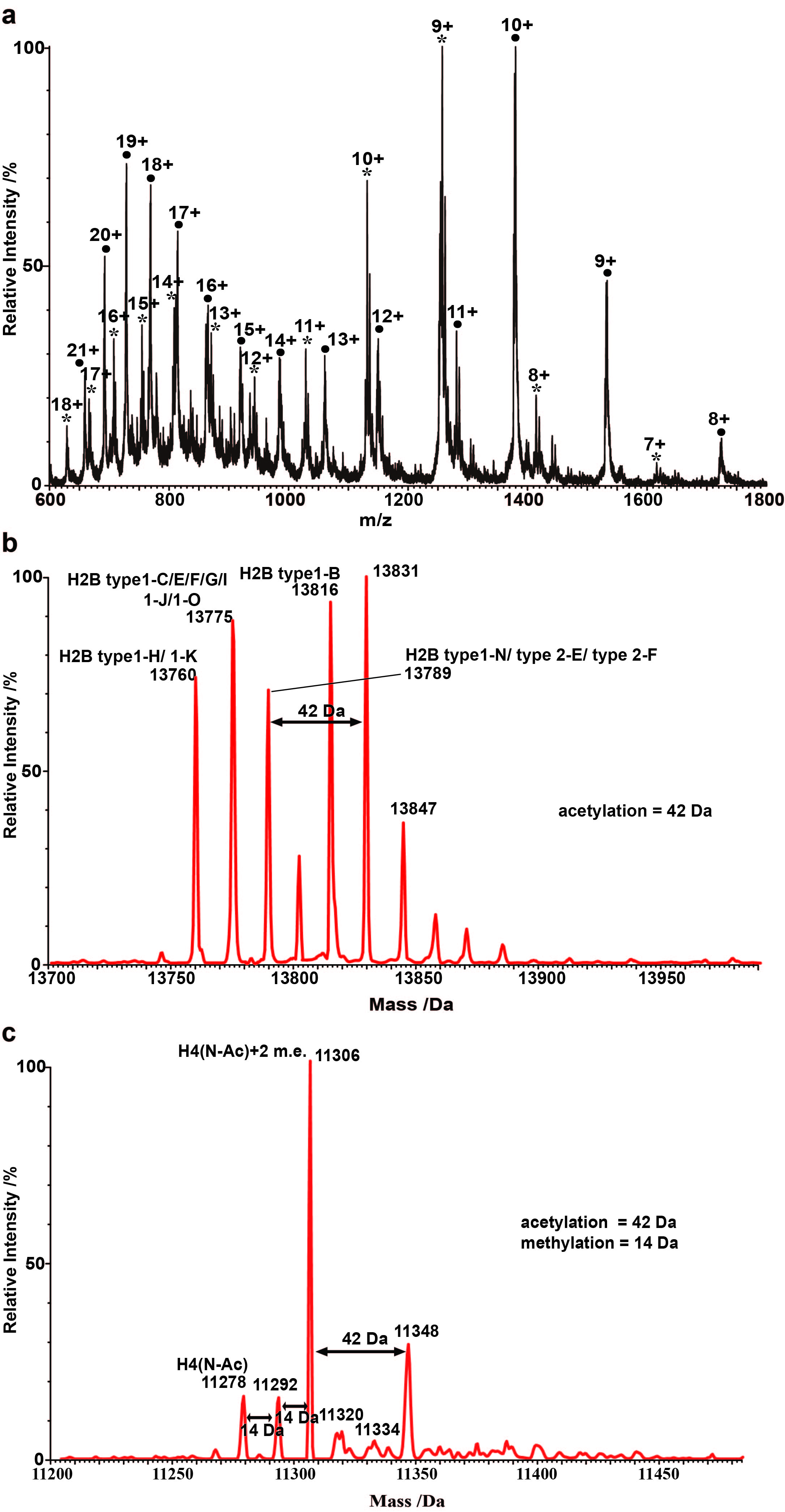

Supplement: Supplemental Material [file KEPI_A_1786305_SM8513.zip › Figure S4.jpg]

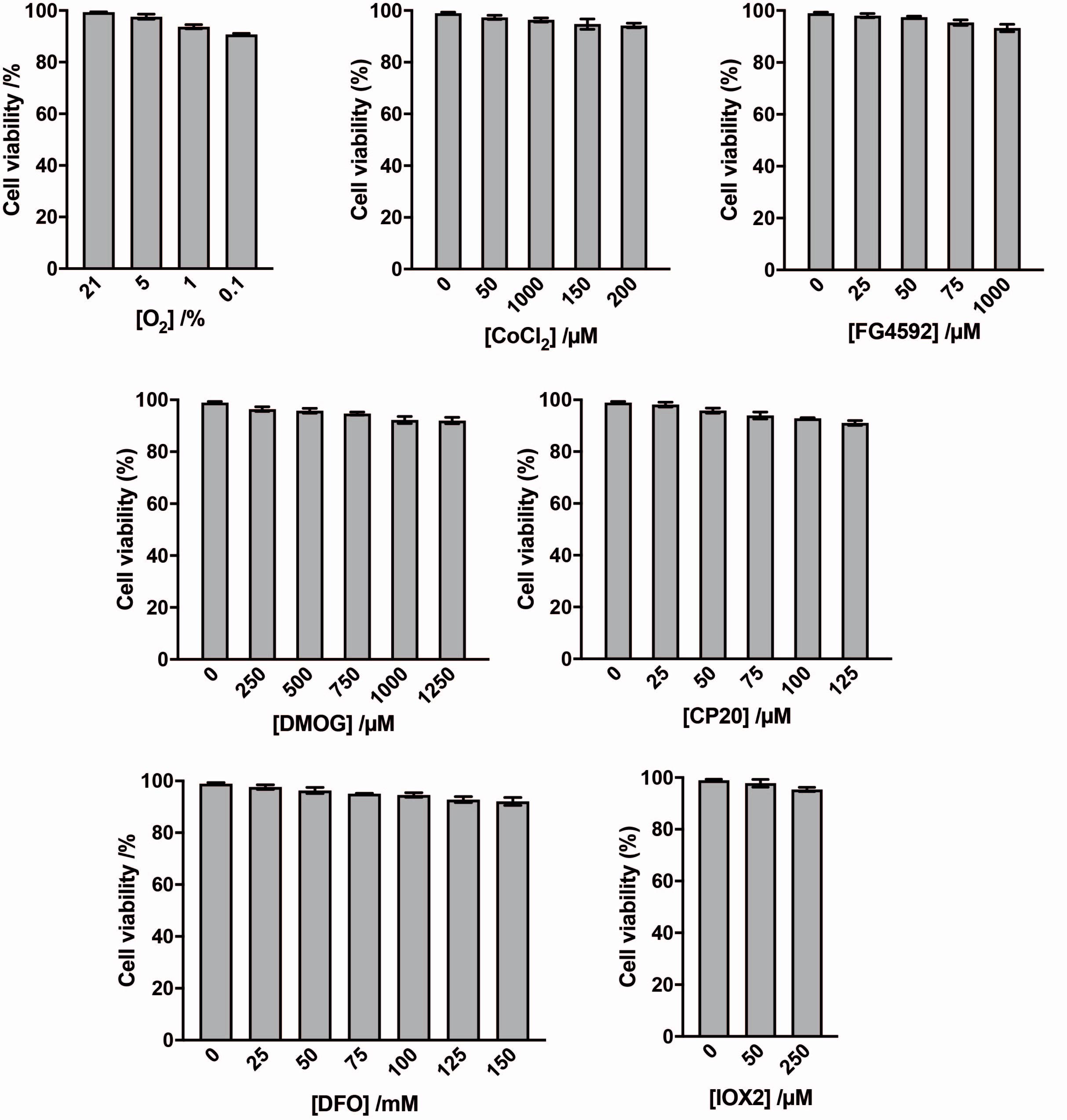

Supplement: Supplemental Material [file KEPI_A_1786305_SM8513.zip › Figure S5.jpg]

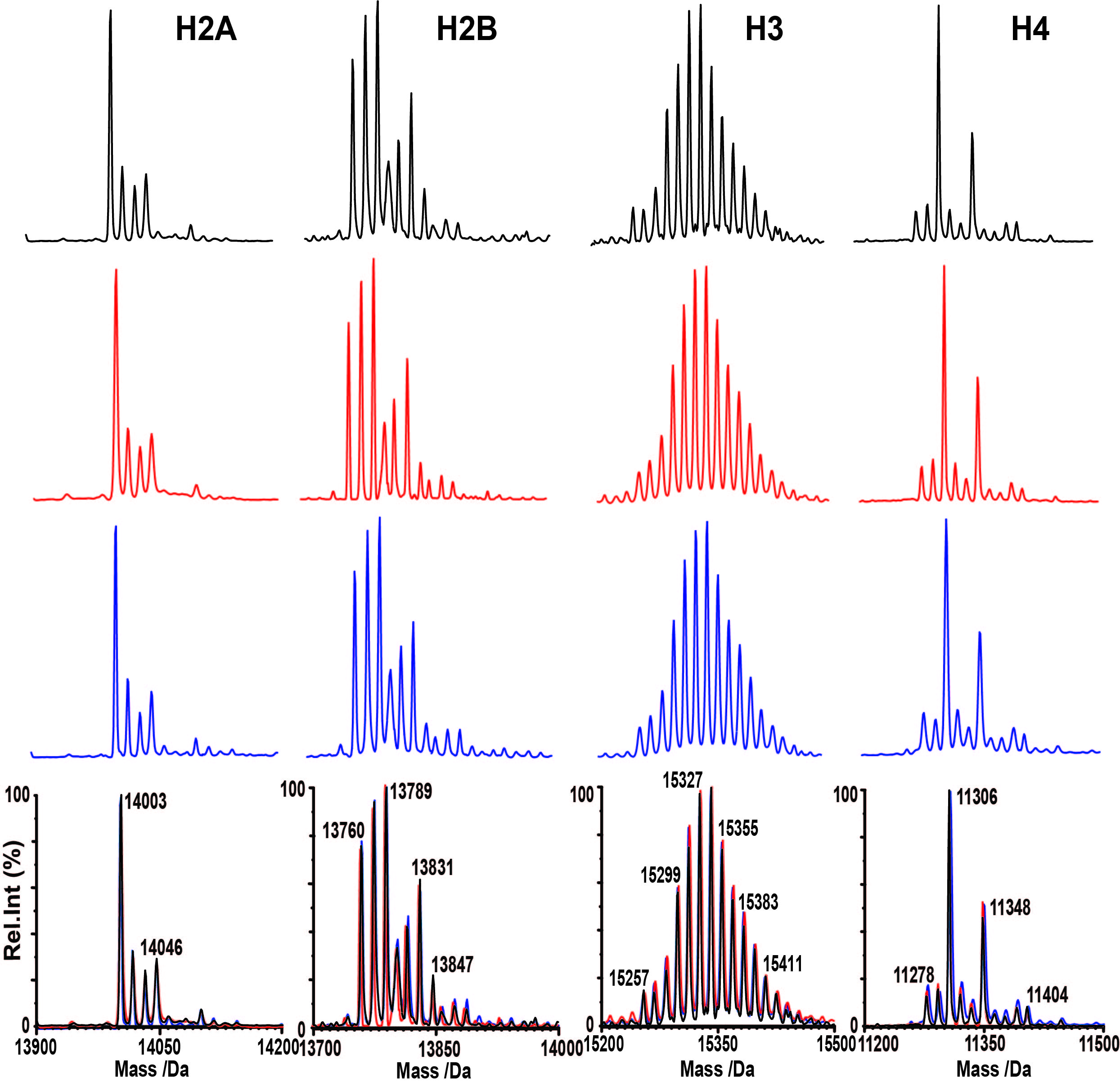

Supplement: Supplemental Material [file KEPI_A_1786305_SM8513.zip › Figure S6.jpg]

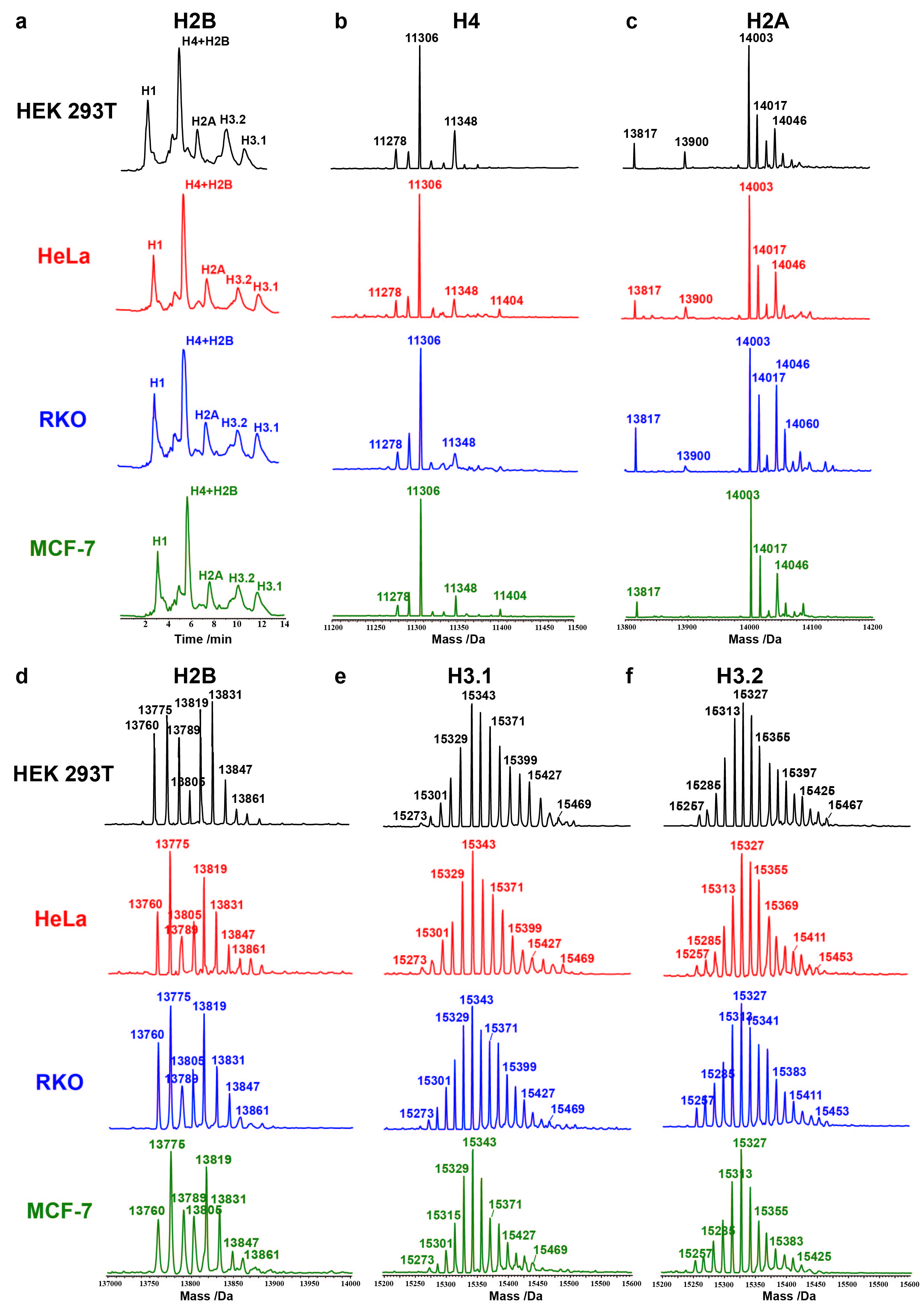

Supplement: Supplemental Material [file KEPI_A_1786305_SM8513.zip › Figure S7.jpg]

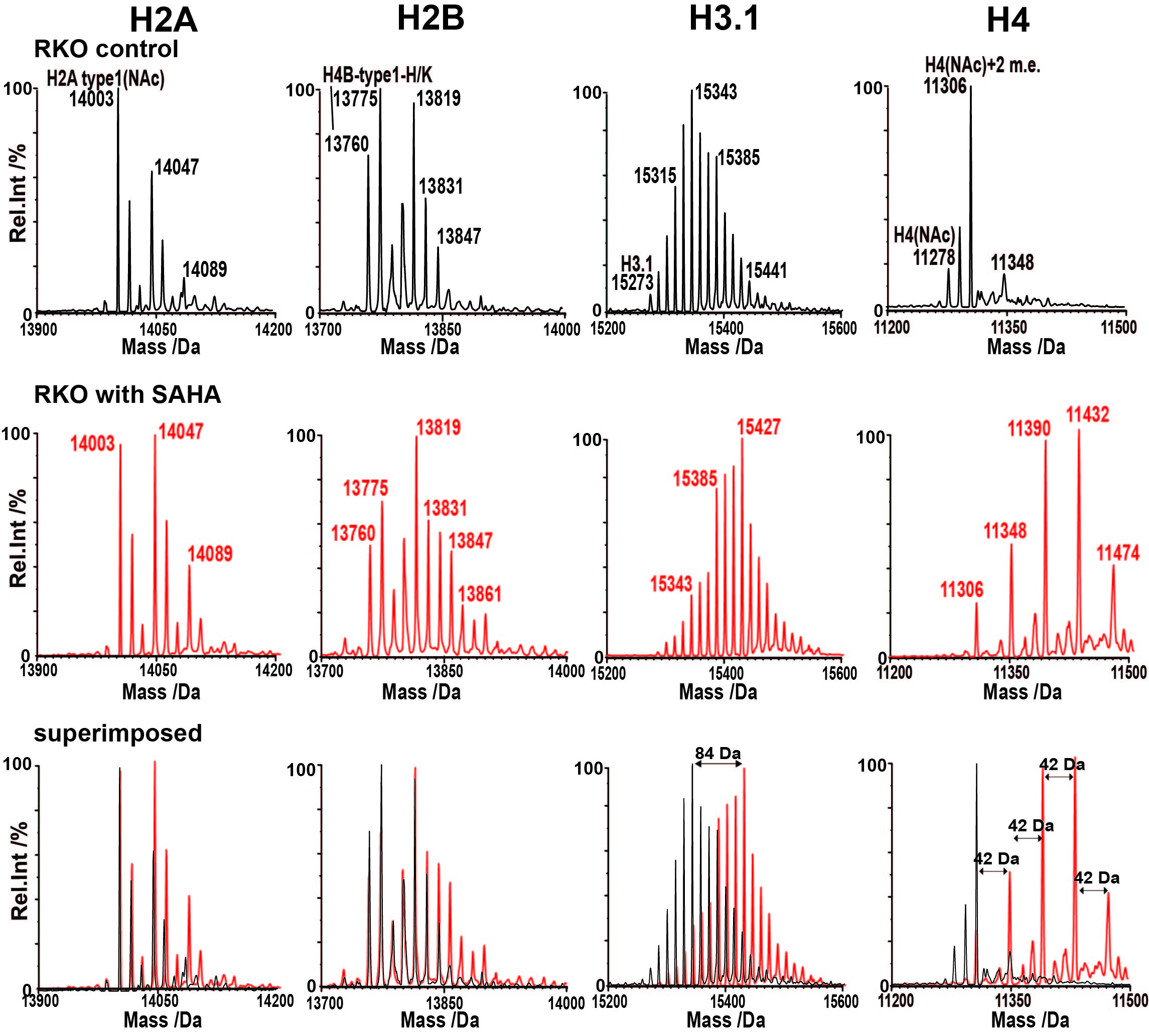

Supplement: Supplemental Material [file KEPI_A_1786305_SM8513.zip › Figure S8.jpg]

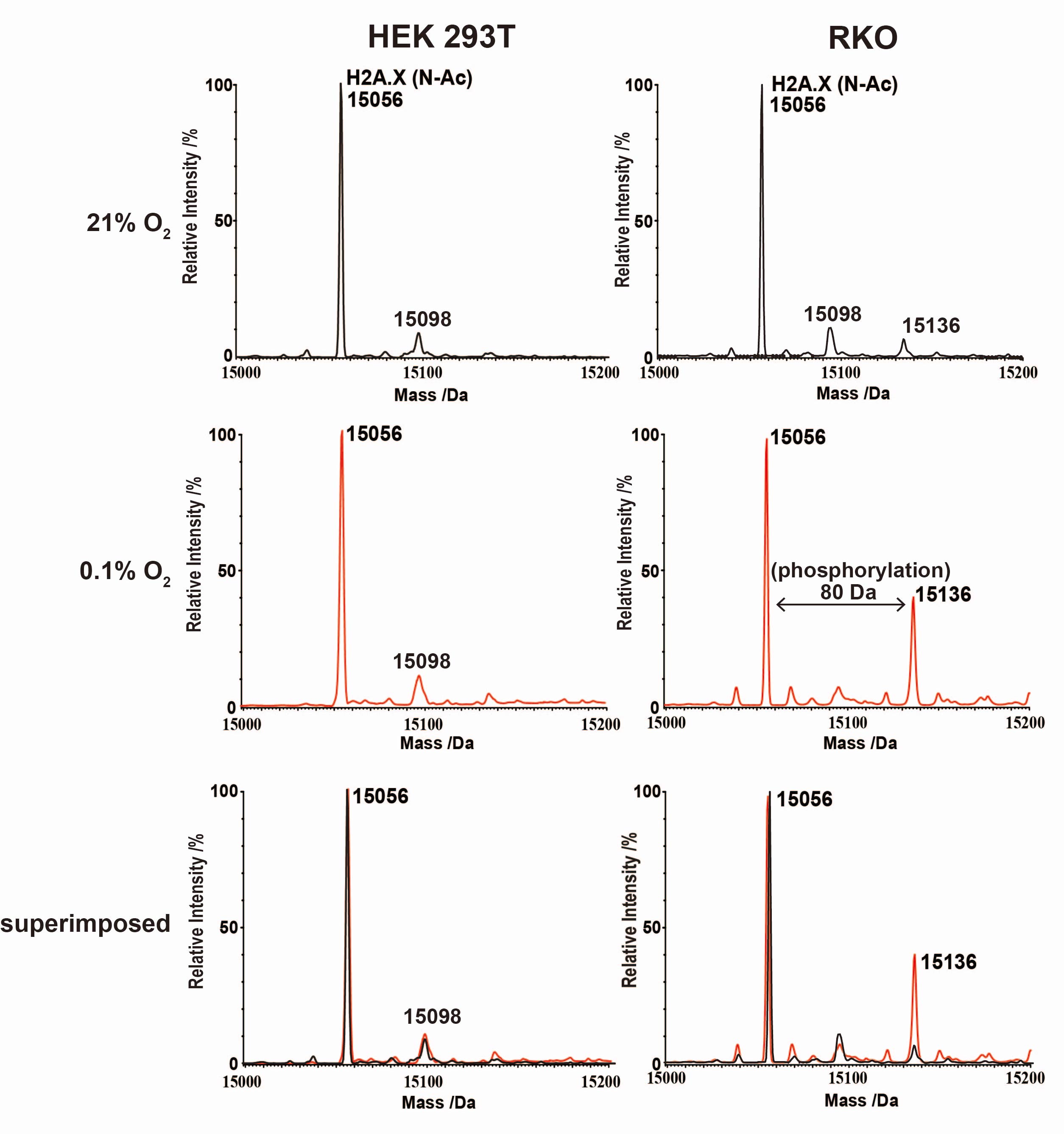

Supplement: Supplemental Material [file KEPI_A_1786305_SM8513.zip › Figure S9.jpg]
